# Preliminary Study on the *In vitro* and *In vivo* Effects of *Asparagopsis taxiformis* Bioactive Phycoderivates on Teleosts

**DOI:** 10.3389/fphys.2016.00459

**Published:** 2016-10-25

**Authors:** Fabio Marino, Gianfranco Di Caro, Concetta Gugliandolo, Antonio Spanò, Caterina Faggio, Giuseppa Genovese, Marina Morabito, Annamaria Russo, Davide Barreca, Francesco Fazio, Andrea Santulli

**Affiliations:** ^1^Department of Chemical, Biological, Pharmaceutical and Environmental Sciences, University of MessinaMessina, Italy; ^2^Department of Veterinary Sciences, University of MessinaMessina, Italy; ^3^Laboratory of Marine Biochemistry and Ecotoxixology, Department of Earth and Sea Sciences, University of PalermoTrapani, Italy

**Keywords:** *Asparagopsis taxiformis*, *Dicentrarchus labrax*, *Sparus aurata*, antibacterial activity, hematological parameters, oxidative stress, toxicity

## Abstract

Several compounds from marine organisms have been studied for their potential use in aquaculture. Among the red algae, *Asparagopsis taxiformis* is considered one of the most promising species for the production of bioactive metabolites with numerous proposed applications. Here, the *in vitro* antibacterial activity, the easy handling and the absence of adverse effects on marine fish species are reported. Depending on the seasonal period of sampling, ethanol extracts of *A. taxiformis* exhibited significantly different inhibitory activity against fish pathogenic bacteria. The extract obtained in late spring showed strong antibacterial activity against *Aeromonas salmonicida* subsp. *salmonicida, Vibrio alginolyticus*, and *V. vulnificus*, and moderate activity against *Photobacterium damselae* subsp. *damselae, P. damselae* subsp. *piscicida, V. harveyi* and *V. parahaemolyticus*. Sea bass and gilthead sea bream were fed with pellets supplied with the alga and algal extracts. The absence of undesired effects on fish was demonstrated. Hematological and biochemical investigations allowed to confirm that the whole alga and its extracts could be proposed for a future application in aquaculture.

## Introduction

In the last decades marine organisms have been considered as potential and promising sources of pharmaceuticals (Newman et al., 2003; Blunt et al., 2008; Mayer et al., 2011). Particularly, seaweeds are considered as a source of bioactive metabolites characterized by a wide spectrum of effects (Fouladvand et al., 2011). Compounds with immunostimulant, cytostatic, antiviral, anti-helminthic, antifungal, antibacterial activities have been detected in all phyla of macroalgae (Ballesteros et al., 1992; Val et al., 2001; Smit, 2004; Talarico et al., 2004; Cumashi et al., 2007; Shanmughapriya et al., 2008; Dhargalkar and Verlecar, 2009; Kamenarska et al., 2009; Vallinayagam et al., 2009; Allmendinger et al., 2010; Bouhlal et al., 2010; Chanda et al., 2010; Vonthron-Sénécheau et al., 2011; Barman et al., 2013; de Jesus Raposo et al., 2015).

Among red algae, *Asparagopsis* species (Bonnemaisoniales, Rhodophyta) showed potent antifungal, antibacterial, and antiprotozoal attributes (Burreson et al., 1975; McConnell and Fenical, 1977; Bansemir et al., 2006; Salvador et al., 2007; Genovese et al., 2009, 2012; Jiao et al., 2011; Manilal et al., 2012). The genus *Asparagopsis* produces several halogenated compounds, such as haloforms, methanes, ketones, acetates, and acrylates, which are assumed as bio-active compounds (McConnell and Fenical, 1977; Woolard et al., 1979; Bansemir et al., 2006). In general, the production of biologically-active metabolites is linked to the ability to store them into specialized storage structures in order to avoid autotoxicity (McKey, 1979). Members in the family Bonnemaisoniaceae, to which *Asparagopsis* species belong, form “vesicle” or “gland cells” (Wolk, 1968; Young, 1977; Womersley, 1998; Paul et al., 2006). The pungent aroma of these algae is due to an essential oil composed mainly of bromoform with smaller amounts of other bromine, chlorine, and iodine-containing methane, ethane, ethanol, acetaldehydes, acetones, 2-acetoxypropanes, propenes, epoxypropanes, acroleins, and butenones, stored in vacuoles within gland cells (Burreson et al., 1976; El-Baroty et al., 2007). *Asparagopsis taxiformis* (Delile) Trevisan de Saint-Léon occurs along tropical and warm temperate coasts, and shows disjoint Atlantic, Mediterranean, and Indo-Pacific populations. It exhibits a strong invasive behavior and therefore it was included in the list of the “Worst Invasives in the Mediterranean Sea” (Zenetos et al., 2010). Recent phylogeographic approaches have shown that this species consists of a number of cryptic species, as well as many other marine algae (Andreakis et al., 2015). At present, *Asparagopsis* species are cultivated for dermo-cosmetical and parapharmaceutical purposes in Atlantic Europe, especially in France, Portugal, Ireland, as well as in Hawaii, Indonesia, Philippines, and New Zealand (Kraan and Barrington, 2005; Mata et al., 2012). Biomass yield proved to be high in nutrients rich water, such as the effluents of fish farms, where the alga acts as a biofilter in integrated multi-trophic aquaculture (IMTA, Barrington et al., 2009).

Natural marine products, including those of algal origin, have enormous pharmacological potentialities, especially when addressing the increasing phenomenon of bacterial antibiotic resistance and the collateral effects that synthetic drugs have both on humans and environment. Although several studies focused on the antimicrobial properties of macroalgae, few studies reported on bacterial pathogens relevant in aquaculture (Val et al., 2001; Liao et al., 2003; Bansemir et al., 2006).

Since several studies underlined the variability in the production of antimicrobials among species within an algal genus, we proposed to use DNA labels to identify unambiguously the algal populations used in applied research.

To verify positive or adverse effects of natural substances, both *in vitro* and *in vivo* trials are needed prior to approach large scale applications in aquaculture. As concerns the *in vivo* studies, hematological profile is an important parameter to be considered as pathophysiological indicator of the whole body status.

The maintenance of a constant volume is a homeostatic requirement in animal cells, when exposed to osmotic gradients inducing a temporary haemodiluition or haemoconcentration. Often the cell volume is compromised by the variation in the concentration of intracellular osmolytes, as a consequence of different processes (e.g., secretion, ion gradients, accumulation of nutrients, cell growth, and proliferation), and a variety of pathologic conditions. Cell volume regulation following cell-swelling, due to osmotic influx of water, involves the release of organic solutes, such as amino acids and ions, through the activation of K^+^ channels and/or anion channels, KCl cotransport, or parallel activation of K^+^/H^+^ exchange and Cl^−^/HCO^3−^ exchange, and the osmotically obliged water efflux (Faggio et al., 2011). This regulatory response is called “regulatory volume decrease” (RVD). Volume-regulatory behavior has been observed in: cells isolated from digestive glands of *Mytilus galloprovincialis* (Torre et al., 2013), *Gobius niger* enterocytes (Trischitta et al., 2004), *Anguilla anguilla* enterocytes (Trischitta et al., 2005), turbot hepatocytes (Ollivier et al., 2006), Ehrlich ascites tumour cells (Hoffmann, 1984), and mammalian red blood cells (Ellory et al., 1985) exposed to osmotic stress.

The work was finalized to investigate: (i) the *in vitro* antibacterial activity of extracts obtained from a DNA barcoded population of *A. taxiformis* collected from the Straits of Messina against fish pathogens, and (ii) the possible effects of dietary administration of the whole alga and its crude extracts on sea bass and gilthead sea bream, by evaluating the RVD of fish hepatocytes, the cytotoxic effects of algal extracts on both red blood cells and isolated hepatocytes of fish, the hematological profiles and the erythrocytes response to oxidative stress.

## Materials and methods

### Algae sampling, identification and processing

Plants of *A. taxiformis* were collected from a marine site (38°12′3.28″N, 15°33′35.63″E), along northeastern Sicilian coast of the Straits of Messina (Italy) in April (ASP001), May (ASP002), June (ASP003), and July (ASP004) 2012-2013, throughout the period in which the alga is present in the field. Fresh plants were washed in sterile seawater, manually cleaned of epiphytes and frozen at −20°C (for the use within few days) or lyophilized (for longer use).

A subsample of each collection was used to obtain COI-5′ sequences according to standard DNA barcoding protocols for red algae (Manghisi et al., 2010). In details, DNA extraction was performed by a Proteinase K protocol and the COI-5′ region was PCR amplified using the primers GazF1 and GazR1 (Saunders, 2005).

Sequencing reactions were performed by an external company (Macrogen Europe, The Netherlands). Forward and reverse sequence reads were assembled with the software ChromasPro (v. 1.41, Technelysium Pty Ltd).

A multiple sequence alignment was constructed in MacClade 4.0 (Maddison and Maddison, 2000), including sequences of *Asparagopsis* species downloaded from GenBank, and subjected to distance analysis in PAUP^*^ 4b10 for the Macintosh (Swofford, 2002) to define species attributions.

For crude extract preparation, frozen or lyophilized plants of *A. taxiformis* were soaked in absolute ethanol at room temperature for 48 h. Extracts were dried with a Rotavapor® at low temperature (35°C) to preserve volatile compounds from evaporation.

Ethanol extracts obtained in April (ASP001), May (ASP002), June (ASP003), and July (ASP004) were used for the *in vitro* antibacterial and cytotoxicity assays, while both ethanol extracts and lyophilized plants were used for the *in vivo* trials.

### *In vitro* experiments

#### Antibacterial activity

Ethanol extracts of *A. taxiformis* (ASP001, ASP002, ASP003, and ASP004) were tested against the following lab strains of fish and shellfish pathogenic bacteria: *Aeromonas salmonicida* subsp. *salmonicida* (13M), *Photobacterium damselae* subsp. *damselae* (Pdd), *P. damselae* subsp. *piscicida* (Pdp), *Vibrio alginolyticus* (Ag37), *V. harveyi* (G5), *V. parahaemolyticus* (L12G), and *V. vulnificus* (OPV10) which are part of the Culture Collection housed at the Dept of Chemistry, Biology, Pharmaceutical and Environmental Sciences of the University of Messina (Italy). The strains were previously isolated and identified as reported in Gugliandolo et al. (2008, 2011). They were maintained at −20°C in Tryptone Soy Broth (Difco) supplemented with 1% (w/v) NaCl (TSB1) and 50% (v/v) glycerol.

Antibacterial activity was evaluated by using the standard disk diffusion method (Kirby Bauer test), as accepted by the National Committee for Clinical Laboratory Standards (NCCLS 2000) and commercially available disks (6 mm in diameter, Oxoid). Strains were overnight grown onto plates of Tryptone Soya Agar (TSA) (Oxoid) amended with 1% of NaCl (TSA1), for 24 h at 25°C. Each strain was suspended in 3 ml of 0.9% NaCl solution with a turbidity optically comparable to that of the 0.5 McFarland standard (1.5 × 10^8^ bacteria/ml) and suspensions (100 μl) were inoculated onto triplicate plates of TSA1.

Each extract (100 mg, equivalent to 3 g of lyophilized algal sample) was dissolved in 1000 μl of absolute ethanol, and 20 μl were applied to sterile filter paper disks. After solvent evaporation, the disks (containing 2 mg of the extract) were placed onto the inoculated plates. Disks soaked with ethanol and submitted to evaporation were used as negative control, and those containing chloramphenicol (30 μg) and tetracycline (30 μg) (Difco, Becton Dickinson and Company, USA) were used as positive control. Plates were incubated overnight at 25°C. The diameter of complete inhibition zone was measured and means and standard deviations (*n* = 3) were calculated. Inhibition zones ≥15 mm in diameter were stated as strong, from 9 to 14 mm as moderate and ≤8 mm as weak in terms of activities (Bansemir et al., 2006).

Minimum inhibitory concentration (MIC) values were determined for the most active extracts, by using the serial dilution assay, as accepted by the European Committee for Antimicrobial Susceptibility Testing (EUCAST) of the European Society of Clinical Microbiology and Infectious Diseases (ESCMID) (2003). Serial dilutions of each extract (16, 8, 4, 2, 1, 0.5, and 0.25 mg/ml) were prepared in tubes of TSB1 and then inoculated with suitable aliquots of overnight cultured bacterial strain in TSB1. To verify the inhibition activity of the extract, 100 μl from all tubes without visible bacterial growth were plated onto TSA1, and incubated overnight at 25°C.

#### Isolation of *dicentrarchus labrax* hepatocytes and regulation volume decrease (RVD) experiments

Ten *Dicentrarchus labrax* specimens (total length 23 ± 2.6 cm; weight 143 ± 10.2 g): control basal diet and ten specimens fed diets enriched with algal ethanolic extracts (total length 20 ± 1.6 cm; weight 100 ± 4.3 g) for 2 weeks were sacrificed with an overdose of MS222 on 0.7 g/l ratio. The liver was removed and subsequently hepatocytes were isolated by collagenase (type IV-activityP125 CDU/mg; CDU = collagenase digestion units, Sigma Aldrich, St. Louis, MO, USA) digestion methods, which required perfusion of the portal vein as described previously (Ollivier et al., 2006). Isolated hepatocytes were maintained in a 370 mOsm/kg physiological saline solution: (millimoles/l): 172 NaCl, 3.4 KCl, 0.8 MgSO_4_, 1.5 CaCl_2_, 5 NaHCO_3_, 0.33 Na_2_HPO_4_, 0.44 KH_2_PO_4_, 5 glucose and 10 Hepes (pH 7.4 and 18°C). Afterwards, the suspension was filtered by 200 μm and 75 μm nylon filters. The cells were suspended again in physiological saline solution and washed twice by centrifugation (1700 g/10 min/4°C). Prior to the experiments the cells were maintained under slight agitation for at least 1 h at 18°C.

In order to immobilize cells, they were seeded onto the bottom of a thermostated Plexiglas chamber (18°C) coated with poly-L-lysine (0.01%) and left to adhere for 2 min on slide. For RVD experiment the isolated cells were visualized and measured by the method described in a previous paper (Faggio et al., 2011). One drop of cell suspension (5 × 106 cells ml^−1^), was placed on a glass slide pretreated with poly-lysine to facilitate cell adhesion (to fix the cells). Two thin strips of double-sided adhesive were placed at the upper and lower edges of the glass slide to support the cover slip and to create an interspace in which the hypotonic experimental solution was then added (millimoles/l: 124 NaCl, 3.4 KCl, 0.8 MgSO_4_, 1.5 CaCl_2_, 5 NaHCO_3_, 0.33 Na_2_HPO_4_, 0.44 KH_2_PO_4_, 5 glucose, and 10 Hepes; pH 7.4). Hepatocyte images, obtained using a CCD camera (DVC Canada color, 1300, 12 bit cooled, slow scan) connected to the microscope were captured at 60 s intervals during 3 min before and 1 min after initiating the hypotonic shock, thereafter they were captured every 5 min for 60 min. Cell images were digitized using a color video camera (Sony). For each slide six/seven cells were counted. The cell areas for each experimental condition (Aexp) were compared to the areas measured in isotonic solution (Ai) at the beginning of the experiment. Consequently, the data were reported as relative area (Aexp/Ai).

#### Cytotoxicity assays

In order to verify if algal extracts administered to fish exert cytotoxic effects, 100 isolated hepatocytes of *D. labrax* (fed diet enriched with algal extract) were measured to verify the viability which was measured by the Trypan blue exclusion method and compared to control group, constituted of cells isolated from control fish (Matozzo et al., 2016). The quality of their lysosomes was evaluated by neutral red retention assay (NR) (which measures the stability of lysosomal membrane). The dye retention was evaluated after an incubation period of 30 and 60 min, according to Repetto et al. (2008). The lysosomes of vital hepatocytes were stained red. All experiments were carried in triplicates.

### *In vivo* trials

The *in vivo* study was carried out at Centre for Experimental Fish Pathology of Sicily (C.I.S.S.), Establishment for Users recognized by the Italian Ministry of Health for experimental activity on aquatic organisms (according to D.L. 26/2014), at the Department of Veterinary Sciences, University of Messina. Fish species used in this study were *D. labrax* and *S. aurata* and all experimental procedures were performed in compliance with EU Directive 63/2010 and D.L. 26/2014 after project evaluation by the competent authorities (authorization n. 587/2016).

#### Pellets preparation for fish feeding

Lyophilized *A. taxiformis* samples, collected from April to July, or its ethanolic extracts were daily administered to fish by feeding them with commercial feed (Skretting) supplemented in lab. Control pellets and pellets amended with alga or algal extracts, were prepared by adding each compound to the ingredients of the basal diet. The uniform distribution of the ingredients in the feed was obtained by mixing pellets with water through magnetic stirrers Heating (VELPA-Scientifica) at 70°C and 4 Stirrer, until obtaining a damp mixture with soft consistency, and subsequently adding algae or algal extracts to the enriched pellet and continuing to mix the mixture at 7 Stirrer for 30 min. The obtained compound was dried at room temperature for 24 h. The feed was then minced and sieved to produce a crumble size suitable to sea bass and gilthead sea bream feeding. The dosage used was 1 g algae/10 g feed and 0.1 g extract/1 g feed.

Different experimental cycles, summarized in Tables 1, 2, were carried out on both adult fish and juveniles to verify possible adverse effects on fish growth obtain enough data to achieve statistically significant results.

**Table 1:** **Sea bass experimental groups submitted to the ***in vivo*** trials**.

**Group**	**Species**	**Number**	**Weight**	**Length**	**Supplementation**	**Duration**
G.0	*Dicentrarchus labrax*	20	15 g	10 cm	Algae	2 months
					1 g/10 g feed	
G.1	*Dicentrarchus labrax*	20	15 g	10 cm	Control	2 months
G.2	*Dicentrarchus labrax*	20	50 g	14 cm	Algae	2 months
					1 g/10 g feed	
G.3	*Dicentrarchus labrax*	20	50 g	15 cm	Algal extract	2 weeks
					0.1 g/1 g feed	
G.4	*Dicentrarchus labrax*	20	50 g	16 cm	Control	2 months
G.5	*Dicentrarchus labrax*	20	100 g	20 cm	Algal extract	2 weeks
					0.1 g/1 g feed	
G.6	*Dicentrarchus labrax*	20	100 g	20 cm	Control	2 weeks

**Table 2:** **Sea bream experimental groups submitted to the ***in vivo*** trials**.

**Group**	**Species**	**Number**	**Weight**	**Length**	**Supplementation**	**Duration**
G.7	*Sparus aurata*	20	7 g	5 cm	Algae	2 months
					1 g/10 g feed	
G.8	*Sparus aurata*	20	7 g	5 cm	Algal axtract	2 weeks
					0.1 g/1 g feed	
G.9	*Sparus aurata*	20	7 g	5 cm	Control	2 months
G.10	*Sparus aurata*	20	50 g	14 cm	Algae	2 months
					1 g/10 g feed	
G.11	*Sparus aurata*	20	50 g	14 cm	Algal axtract	2 weeks
					0.1 g/1 g feed	
G.12	*Sparus aurata*	20	50 g	14 cm	Control	2 months

Each group of adult Mediterranean sea bass and juveniles were reared in 500 l tanks, whereas gilthead sea bream, coming from a local commercial fish farm, were reared in 100 l tanks. Each tank was supplied by an independent biological and mechanical filtration system, artificial aeration and illumination with neon to assure perfect conditions of animal welfare. The water was prepared using a synthetic salt (Blue treasure, Sea Salt) obtained by mixing pure elements to obtain marine water chemically and biologically excellent. Water parameters in all tanks were checked daily by a multiparametric probe (HI9829 Aquaprobe, Hanna instruments, Padova, Italy) and the following values were maintained for all the 2 months of experiments: temperature 20–22°C; salinity 30%0; pH 8~. Moreover, a photoperiod of 12 h light/12 h dark was provided. In the first trial 80 sea bass subdivided in 4 fish groups were used.

G0. 20 juvenile Mediterranean sea bass, of about 6 months in age, with a median weight of 15 g and a median length of 10 cm, were randomly selected by a pool of healthy subjects coming from a local commercial fish farm; after quarantine, fish were successively transferred to the experimental tank for the trial and were fed with pellet supplied with algae.

As a control group, further 20 age-matched (G1) specimens were used and fed with commercial pellet. G5. 20 adult Mediterranean sea bass (*D. labrax*), 12 months aged, median weight 100 g, 20 cm in length, were fed with pellets supplied with algal extract. Water parameters as previously referred.

G6. 20 sea bass fed with control pellet.

All the experimental groups were treated respectively for a 8 weeks (G0) and 2 weeks (G5 and G6) long period. The shorter time of the trial with algal extract was related to initial appearance of adverse effects on behavior which suggested to anticipate the sacrifice according to general guidelines for animal welfare during toxicological studies.

Water parameters and health status of fish, as well as their vitality and appetite, were daily checked and all possible variations were registered.

In the second experimental cycle, 60 gilthead sea bream, coming from a local commercial fish farm, were subdivided in three groups (G7–G9) each constituted of 20 fish, median weight 7 g and a median length of 5 cm. G7 was supplied with algae, G8 with algal extract, G9 was used as age-matched normal control. The third experimental trial was performed repeating the feed protocols already described using sea bass and sea bream alevins. A total of 120 fish (60 sea bass and 40 sea bream) 50 g in medium weight were used subdivided in groups of 20. Three groups of sea bass (G2 alga, G3 algal extract, G4 normal control) and 3 groups of sea bream (G10 alga, G12 control, G11 algal extract).

Fish were constantly monitored for a period of 10 min after feed administration to evaluate feed palatability and effective food consumption.

#### Sample collection

Blood samples were obtained before and at the end of each trial on both treated fish and control fish. For hematological analysis only fish ranging in weight from 50 to 100 g were considered. Fish (*n* = 10 for each group) from G 7, 8, and 9 (for *D. labrax*) and from G 10, 11, and 12 (*S. aurata*) were deeply anesthetized with MS222 (0.4 g/l) and successively underwent venipuncture for blood collection. Blood samples were obtained from caudal vein using a 18 G × 1 ½ syringe and collected into micro tubes (Miniplast 0.6 mL, LP Italiana Spa, Milano) containing EDTA (1.26 mg/0.6 mL).

All samples were analyzed in triplicate by the same operator. After the last blood sampling, fish were euthanatized with MS222 (0.7 g/l) and tissue samples were collected from skin, gills, heart, liver, gut, spleen, kidney, and brain for routine histopathology. Briefly, 5 μm thick paraffin sections were obtained and stained with hematoxylin-eosin and Giemsa methods, mounted on slides and observed by light microscope.

#### Hematological analyses

The hematological profile was measured using an automated hematology analyzer (HeCo Vet C, SEAC, Florence, Italy) with a method already used both in *S. aurata* and *D. labrax* (Fazio et al., 2012a,b, 2013, 2015). Hemogram included the white blood cell count (WBC), red blood cell count (RBC), hematocrit (Hct), hemoglobin concentration (Hgb), mean corpuscular volume (MCV), mean corpuscular hemoglobin (MCH), mean corpuscular hemoglobin concentration (MCHC), and thrombocyte count (TC).

#### Erythrocyte oxidative stress

At the end of the feeding period, both *D. labrax* and *S. aurata*, 100 and 50 g in weight respectively were anesthetized and divided into 2 groups (*n* = 8) (control and treated with dietary whole *A. taxiformis*). The blood was collected and inserted in distinct tubes containing EDTA (1.26 mg/0.6 ml). The plasma and erythrocytes were separated by centrifugation at, 1020 g for 5 min and the red blood cells (RBC) were washed with isosmotic solution.

The evaluation of the levels of erythrocytary hemolysis and met-Hb, membrane peroxidation, determination of the total reducing power (FRAP) and dosage of acetylcholinesterase on erythrocyte membrane were carried out according to Ficarra et al. (2013, 2016), Scala et al. (2015) and Carelli-Alinovi et al. (2016). Nitrites and nitrates (as an index of stability of the phospholipidic bilayer), quantization of the total thiols and gluthathione (index of the cells redox state and onset of oxidative injuries) were performed utilizing a commercial determination kit supplied by Sigma Aldrich.

### Statistical analysis

To assess the significant differences in *in vitro* antibacterial activity among the extracts, one-way analysis of variance (ANOVA) was applied by using SPSS software v.17 (SPSS Italia, Rome, Italy).

*In vivo* analytical data, represented as mean (M) ±standard error of the main (SEM), are the averages of three analyses carried out by the same operator. Hematological samples exhibited parallel displacement to the standard curve. The overall intra-assay coefficient of variation was <9%.

Data obtained for different hematological parameters were tested for normality using Kolmogorov-Smirnov test. *P* < 0.05 was considered statistically significant.

One-way analysis of Variance (ANOVA) was used to determinate a statistically significant effect of treatments on all hematological parameters obtained respect to control group for *S. aurata* and *D. labrax*. Bonferroni's multiple comparison test was used for *post hoc* comparison.

Data were analyzed using statistical software prism v. 5.00 (Graphpad Software Ltd., USA, 2003).

## Results

### Algal extracts

Algal specimens were identified using DNA barcoding. Eight COI5′ sequences were generated, one per each collection. All obtained sequences were identical among them and compared to a sample previously sequenced from the same marine site (Genbank accession number JN642177). The population under investigation resulted as *A. taxiformis* “lineage 2,” as named in Andreakis et al. (2007).

Ethanolic extracts and lyophilized plants were produced and provided for *in vivo* and *in vitro* trials. No evaluation of the efficiency of the extraction procedures was performed due to the nature of algae, being the amount of bioactive molecules variable in time in relation to harvest period, site and other variables. This efficiency will be evaluated when bioactive molecules will be purified.

#### *In vitro* experiments

#### Antibacterial activity

No inhibition activity was observed using extracts from algal samples collected in June (ASP003) and July (ASP004), while those collected in April (ASP001) and in May (ASP002) showed antibacterial activities.

Differently from ASP001, ASP002 was active against the whole panel of target pathogens. The inhibition activity of the two extracts was significantly different (*P* < 0.01) as compared to control.

ASP002 (2 mg/disk) showed strong antibacterial activity against *V. alginolyticus* (Ag37) (17.2 ± 1.5 mm), *V. vulnificus* (OPV10) (17.1 mm ± 1.1), and *Aer. salmonicida* (13M) (15.2 ± 1.0 mm), and moderate activity against *P. damselae* (Pdd) (13.8 ± 1.8), *P. piscicida* (Pdp) (13.8 ± 0.8), *V. harveyi* (G5) (12.4 ± 1.0), and *V. parahaemolyticus* (L12G) (12.2 ± 1.3).

ASP001 showed a moderate activity against *V. alginolyticus* (Ag37) (12.8 ± 1.2), *Aer. salmonicida* (13M) (12.5 ± 0.1), and *V. harveyi* (G5) (12.0 ± 1.5).

The MIC values of the ASP002 extract were the following: 4 mg/ml against *Aer. salmonicida* (13M), *P. damselae* (Pdd), and *P. piscicida* (Pdp), and 2 mg/ml against *V. harvey*i (G5).

#### RVD experiments

**Figure 1:**
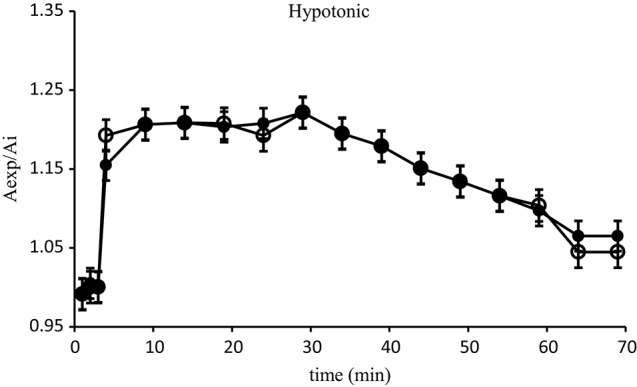
**Relative area changes of the ***D. labrax*** isolated hepatocytes (fish fed with control basal diet) exposed to the hypotonic solution (●) *N* = 10**. Relative area changes of the *D. labrax* isolated hepatocytes (fish fed with diets enriched with algal extract) exposed to the hypotonic solution (o) *N* = 10. Values are means ± S.E.

Isolated hepatocytes of *D. labrax*, fed with control basal diet, exposed to the rapid change of osmolarity, initially increased their size and then tended to return to their initial volume. As showed in Figure 1 the hepatocytes reached their maximum swelling, corresponding to an increase of 15% of volume, after 5 min exposure to the hypotonic solution. Thereafter, they exhibited an RVD response. No significant difference due to fed diets enriched with algae extract was observed.

#### Cytotoxicity assay

The results obtained by Trypan blue exclusion method, supported by the results obtained by Neutral Red (NR), revealed no apparent changes in cellular viability neither in *D. labrax* isolated hepatocytes fed with control basal diet (95% of cellular viability), nor in *D. labrax* isolated hepatocytes fed diets enriched with algal extracts (95% of cellular viability).

#### *In vivo* trials

#### Fish feeding

During administering of all the different specific feeds no adverse effects, such as mortality and pathological symptoms, nor significant modifications of the water parameters were detected. Only when checking the effects of algal extracts, slight behavioral modifications were detected probably due to the higher concentration of the ethanolic extracts in different bioactive molecules, with both positive and negative effects, as compared to the whole algae. Both the remaining specific supplements used, alga and its extracts, did not modify palatability of pellet, although, only at the beginning of the administration, in *S. aurata* a slight delay (less than 2 min) in eating pellet was registered.

Histological results revealed no undesirable changes in all the tissues of the fish studied.

#### Hematological analyses

Mean values ±SEM of hematological parameters obtained in control group and experimental groups for *D. labrax* and *S. aurata* are reported in Tables 3, 4 respectively.

**Table 3:** **Mean values ±SEM of hematological parameters of ***D. labrax*** (*n* = 10 for each group) obtained in Control Group (G4, gray labeled) and Experimental Groups (G2 and G3)**.

**Parameters**	**Control group**	**Experimental groups**	
	**G4**	**G2**	**G3**
RBC (x 10^6^/μl)	3.50 ± 0.22^a^	3.42 ± 0.90^a^	3.48 ± 0.50^a^
Hgb (g/dL)	9.80 ± 0.54^a^	9.27 ± 0.30^b^	9.30 ± 0.54^b^
Hct (%)	41.40 ± 1.20^a^	40.70 ± 1.30^a^	41.50 ± 1.60^a^
MCV (fL)	118.00 ± 2.70^a^	119.00 ± 1.60^a^	119.25 ± 2.00^a^
MCH (pg/cell)	28.00 ± 1.00^a^	26.32 ± 0.80^a^	26.72 ± 1.20^a^
MCHC (g/dL)	23.67 ± 0.90^a^	22.11 ± 0.70^a^	22.41 ± 1.00^a^
WBC (x10^3^/μl)	70.20 ± 3.54^a^	68.40 ± 1.54^a^	67.00 ± 2.10^a^
TC (x10^3^/μl)	54.00 ± 0.54^a^	52.00 ± 0.32^a^	53.20 ± 0.27^a^

Means without the same alphabetical characters within the same parameters represent statistical differences (P < 0.05).

**Table 4:** **Mean values ± SEM of hematological parameters of ***S. aurata*** (***n*** = 10 for each group) obtained in Control Group (G12, Gray labeled) and experimental Groups (G10 and G11)**.

**Parameters**	**Control group**	**Experimental groups**	
	**G12**	**G10**	**G11**
RBC (x 10^6^/μl)	3.77 ± 0.13^a^	3.10 ± 0.11^b^	3.42 ± 0.08^b^
Hgb (g/dL)	11.75 ± 0.29^a^	9.61 ± 0.19^b^	10.67 ± 0.10^b^
Hct (%)	43.62 ± 1.37^a^	35.92 ± 1.01^b^	41.73 ± 0.56^b^
MCV (fL)	116.10 ± 2.65^a^	117.5 ± 3.78^a^	129.40 ± 1.27^b^
MCH (pg/cell)	31.29 ± 0.48^a^	31.20 ± 1.06^a^	31.40 ± 0.68^a^
MCHC (g/dL)	27.07 ± 0.48^a^	26.89 ± 0.60^a^	25.62 ± 0.37^a^
WBC (x10^3^/μl)	74.70 ± 2.15^a^	62.17 ± 3.74^b^	54.97 ± 2.04^b^
TC (x10^3^/μl)	36.70 ± 0.56^a^	33.10 ± 0.83^b^	36.60 ± 1.32^a^

Means with different alphabetical characters within the same parameters represent statistical differences (P < 0.05).

In the control group all hematological parameters were within the physiological range of *S. aurata* and *D. labrax* reported in previous research (Faggio et al., 2014; Fazio et al., 2015).

For *D. labrax* (Table 3) no statistical significant different were observed between experimental groups (G2 and G3) respect to the control group (G4) for all parameters, with the only exception of Hb that showed a significantly reduction in experimental groups (G2 and G3) in the respect to the control group (G4). For *S. aurata* (Table 4) ANOVA analysis showed significant changes in all hematological parameters registered in the experimental groups (G10 and G11) respect to the control group (G12), except for MCH and MCHC. In particular, RBC, Hgb, and Hct values were lower in groups G10 and G11 respect to the control group (G12), while MCV showed an increase in G11 respect to the control group (G12).

The mean values of WBC in fish fed with alga (G10) or algal extracts (G11) were 62.17 × 10^3^ μl and 54.97 × 10^3^μl respectively, which were significantly lower than that registered in the control group (74.70 × 10^3^μl). Fish subjected to administration of alga (G10) showed significantly lower numbers of TC (33.10 × 10^3^μl) than that of the control group (36.70 × 10^3^μl).

#### Oxidative erythrocyte stress

**Figure 2:**
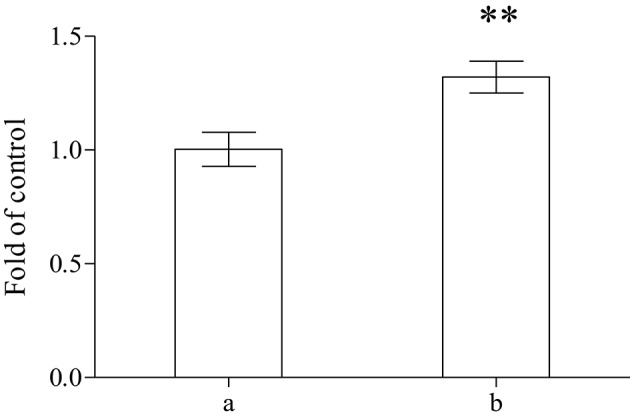
**Determination of FRAP in the plasma performed on control (a) or on specimens of sea bass fed with algae (b) (***n*** = 8)**. ^**^Showed a significant difference *P* < 0.05.

A significant increase of the total reducing capability (FRAP method) of plasma was demonstrated (Figure 2) on sea bass fed for 2 months with the whole *A. taxiformis*. The results obtained with sea bass, that are almost completely superimposible with those obtained with sea bream, are depicted.

**Figure 3:**
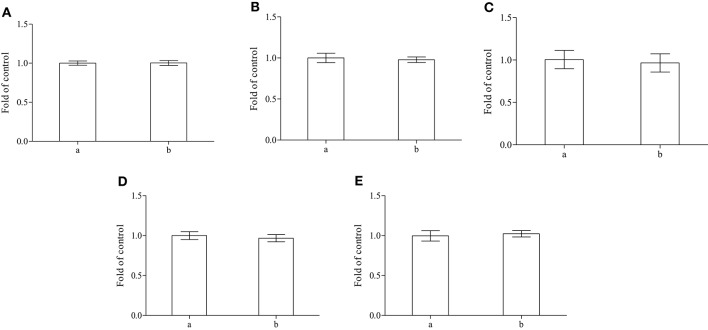
**Determination of lipid peroxidation (A), acetylcholinesterase activity (B) and met-Hb amount (C) in isolated erythrocytes and total thiols in both plasma (D) and cytoplasm of erythrocytes (E) performed on control (a) or on sea bass fed with alga (b) (***n*** = 8)**.

This result seems to be confirmed by the experimental evidences obtained by: (i) the lipid peroxidation tests (Figure 3A), (ii) the trials of integrity of membrane, based on determination of acetylcholinesterase activity (Figure 3B), (iii) the quantification of met-Hb formed (Figure 3C) and (iv) the total thiols contents in both serum and cytoplasm of erythrocytes (Figures 3D,E). In our experiment we did not found significant variation between fish fed with the whole *A. taxiformis* and control fish.

**Figure 4:**
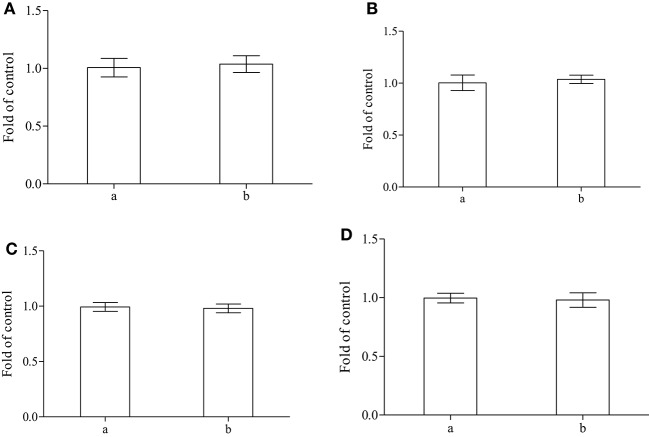
**Determination of reduced glutathione and total thiols in plasma (A,C) and cytoplasm of erythrocytes (B,D) performed on control (a) or sea bass fed with alga (b) (***n*** = 8)**.

When the main endogenous antioxidants were evaluated in fish treated with alga, the amounts of reduced glutathione and of nitrous metabolites showed no detectable variations in both plasma and cytoplasm of erythrocytes (Figures 4A–D).

Thus, fish fed with whole *A*. *taxiformis*, did not show significant alterations in the physiological and biochemical processes analyzed in sea bass and sea bream.

## Discussion

Marine algal compounds represent a source of novel bioactive metabolites with unexpected potentialities. The synthesis of antimicrobial compounds by seaweeds may vary with geographic location, ecological factors, and physiological conditions (active growth or sexual maturity) (Genovese et al., 2012 and reference listed therein).

Depending on the period of sampling, probably due to a different content of biomolecules, ethanolic extracts of *A. taxiformis* exhibited significantly different inhibitory activity against fish pathogenic bacteria. The extract obtained in late spring was active against the whole panel of pathogens, including *P. damselae* subsp. *damselae* and *P. damselae* subsp. *piscicida*, considered resistant to most antibiotics, *V. harveyi*, an emerging pathogen of fish and shrimps, and *V. parahaemolyticus* and *V. vulnificus*, recognized as causative agents of human diseases associated with the consumption of raw shellfish. Seasonal differences in the production of bioactive compounds could be associated with the maximum growth period of algae (Hornsey and Hide, 1974), which actually occurs in late spring for *A. taxiformis* at our latitude. The antibacterial activity (evaluated as inhibition zones and MIC values) of crude extracts here reported against *Aer. salmonicida, P. damselae* subsp. *damselae, P. damselae* subsp. *piscicida* and *V. harveyi* was lower than that of antibiotics used as control. However, future investigations should attempt to purify active compounds from crude extracts and to identify their chemical composition.

The whole alga and its extracts did not show any cytotoxicity effects on fish hepatocytes, confirming our results previously reported (Genovese et al., 2012).

*S. aurata* fed with whole algae and algal extracts showed significant decreased in RBC, Hgb, and hematocrit (%) (Hct) in comparison with those of the control group. These results are in accordance with those for European Sea bass juveniles fed garlic powder diets at dietary inclusion levels of 4 and 6%, reported by Irkin et al. (2014), the only study available to date for a comparison. The numbers of white blood cells (WBC), which have important implications for the immune response and the ability of the animal to fight infection, significantly decreased in both *D. labrax* and *S. aurata* treated with whole algae and algal extracts, as compared to the control group. In *S. aurata*, the results showed a statistically significant lower WBC values in experimental groups (G10 and G11) respect to control group, whereas TC showed statistically significant lower values in G10 respect to G11 and control group. In fish WBC together with TC play an important role for the defense response (Stosik et al., 2001; Kollner et al., 2004; Tavares-Dias and Moraes, 2004; Passantino et al., 2005; Tavares-Dias et al., 2007, 2008; Martins et al., 2008; Prasad and Charles, 2010; Prasad and Priyanka, 2011), therefore these findings suggest a negative effect of algae at this specify dose in body's immune system. Observed negative impacts of algal extracts on the hematological parameters, would lead to discourage the use of such compounds as food supplements. Nevertheless, such negative effect could be related to a dose/response effect, so that it is possible that further trials could detect an hormetic response after lower dose exposure (Calabrese and Baldwin, 2002; Calabrese, 2008). Moreover, both positive and negative effects on total WBC count have been registered in fish fed with different concentrations of *Fusarium moniliforme* (Lumlertdacha et al., 1995).

The individuation of the minimum effective dose will let start the future precompetitive development of the algal extracts for a field evaluation on a wide scale and the full application in aquaculture.

Differently, fish fed with both alga and algal extracts maintained high levels of endogenous antioxidants, evaluated as total amount of reduced glutathione, in serum and in the cytoplasm of erythrocytes, indicating a lower inclination to suffer oxidative damages of all the compounds tested. The presence of bioactive substances in the algal extracts could allow the fish to maintain a high reducing redox state, eliciting a cellular welfare ready to contrast the oxidative injuries.

The supplementation with alga and algal extracts did not modify growth rate (data not shown) and zootechnical performances, even if the short period of the challenges (2 weeks/2 months) did not allow us to definitely assess the nutritional significance of the algal supplements. Further long time investigations are need to clarify the nutritional potential of *A. taxiformis* and its extracts on Mediterranean marine fish species.

Our findings suggest that the whole alga *A. taxiformis* and its extracts, with demonstrated antibacterial activity, could be proposed for future applications in aquaculture, as alternative tool in contrasting most of common fish pathogens. Furthermore, the results obtained could be used to create in the next future a new industrial field for the production of supplements useful for a modern and eco-sustainable fish farming.

## Author contributions

FM and GD were involved *in vivo* experiments; CG and ASp were responsible for *in vitro* antimicrobial evaluation; CF performed cytotoxicity experiments; MM and GG selected algal species for *in vitro* and *in vivo* evaluation; AR and DB carried out the Evaluation of oxidative erythrocyte stress; FF accomplished the hematological analyses, whereas ASa reviewed the manuscript.

## Funding

Project IZS SI 04 2014, Department of Chemical, Biological, Pharmaceutical and Environmental Sciences, University of Messina.

### Conflict of interest statement

The authors declare that the research was conducted in the absence of any commercial or financial relationships that could be construed as a potential conflict of interest.
